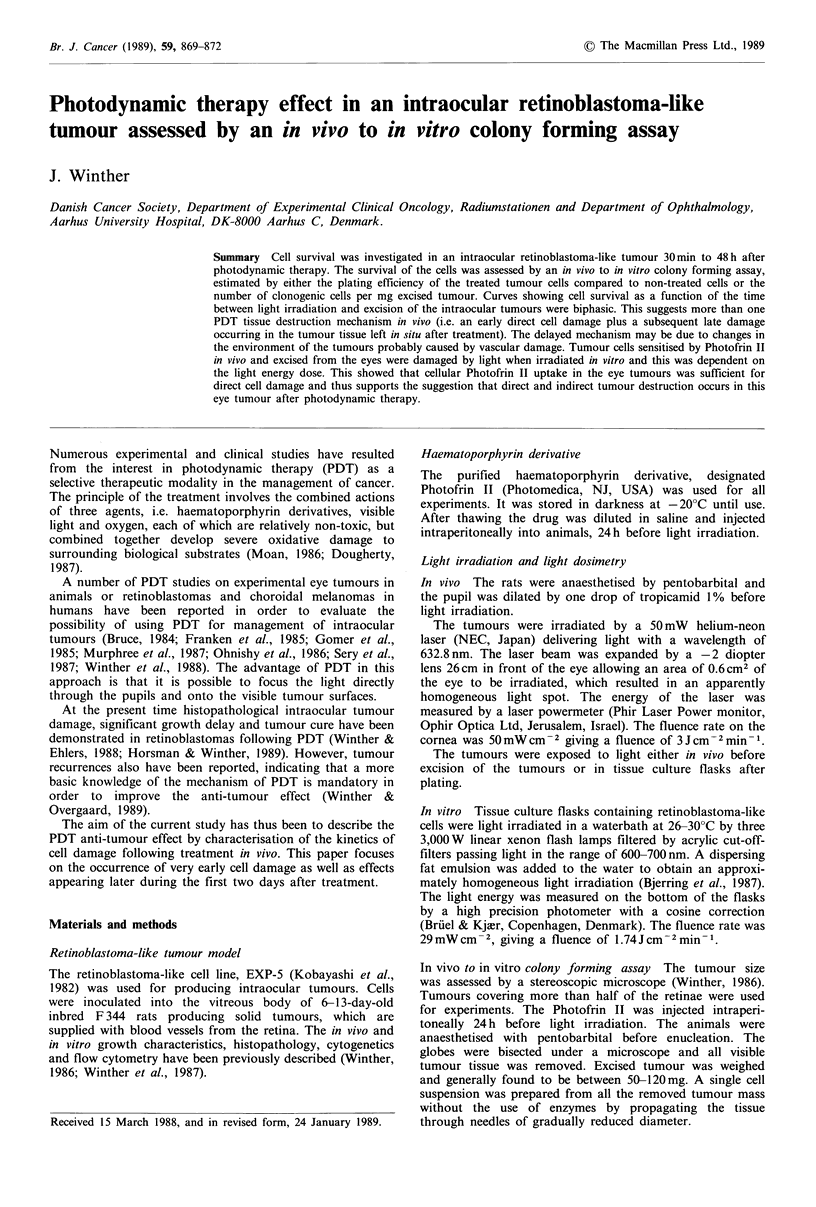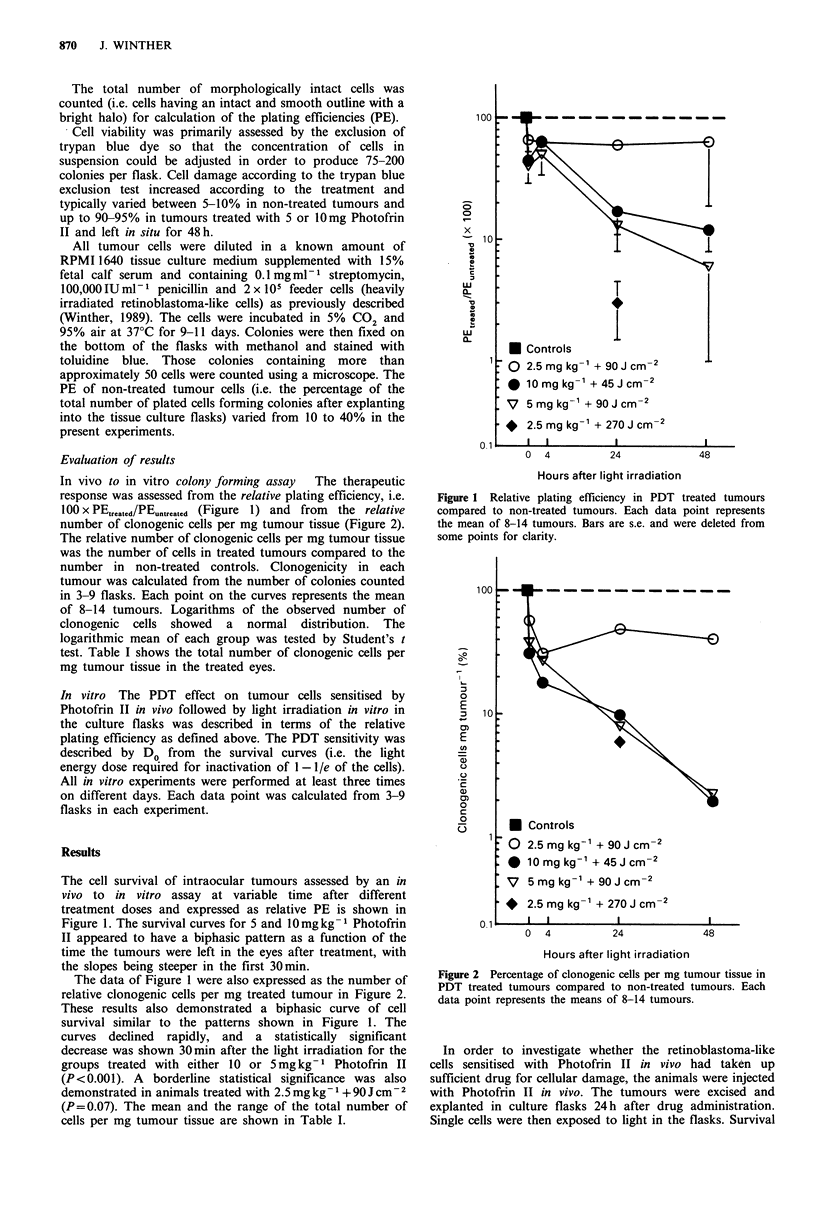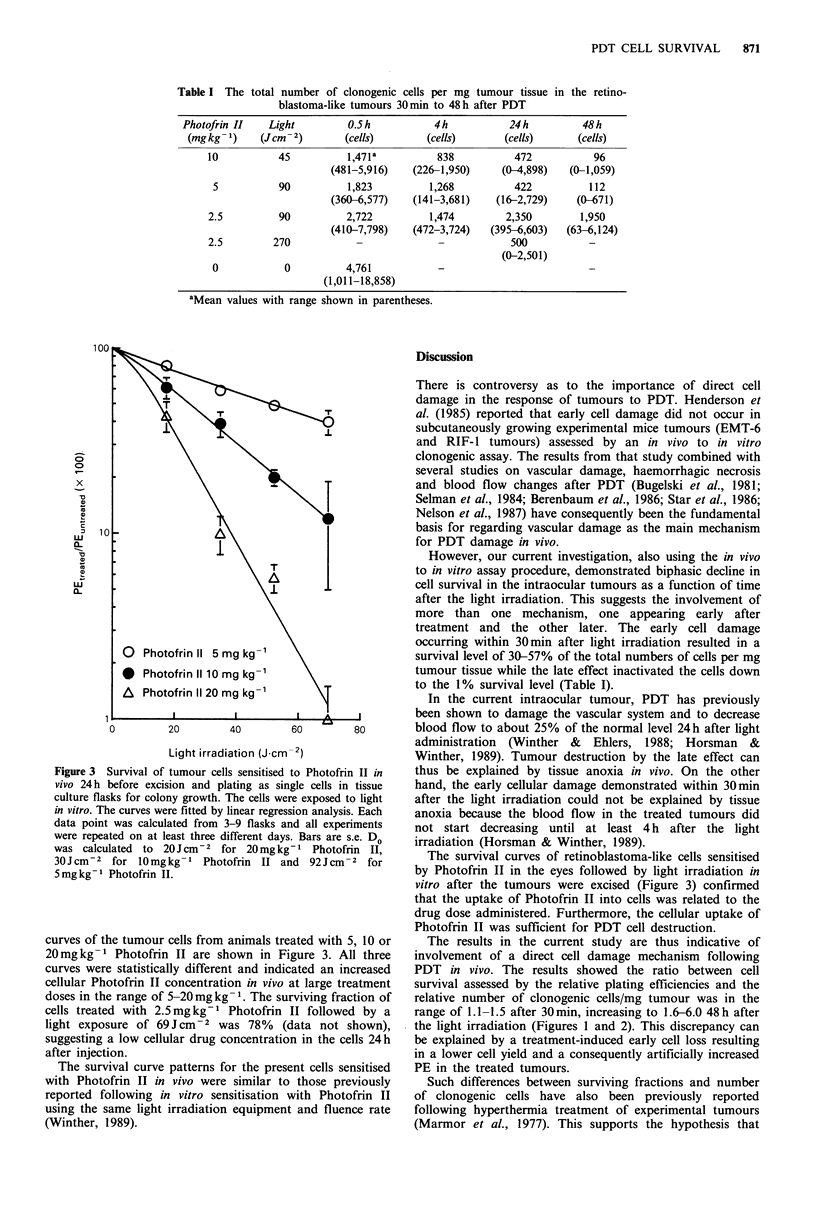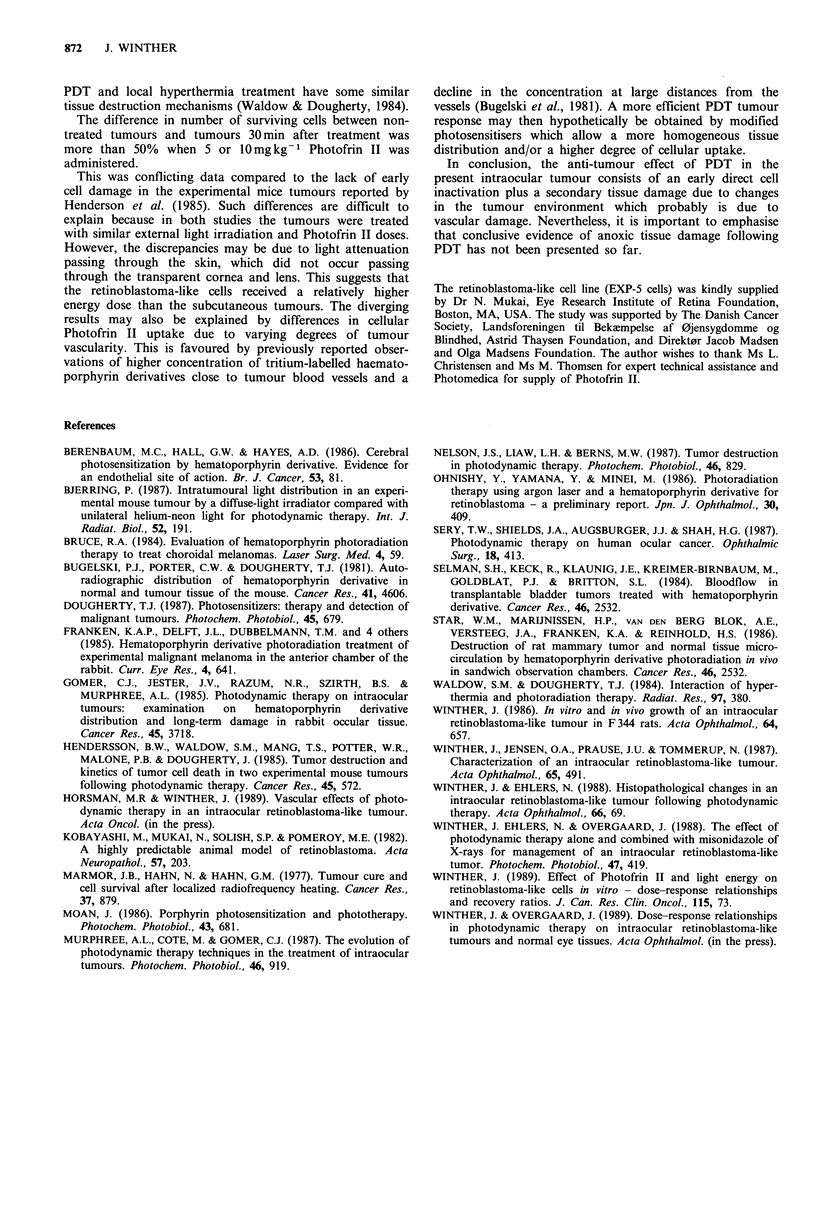# Photodynamic therapy effect in an intraocular retinoblastoma-like tumour assessed by an in vivo to in vitro colony forming assay.

**DOI:** 10.1038/bjc.1989.184

**Published:** 1989-06

**Authors:** J. Winther

**Affiliations:** Danish Cancer Society, Department of Experimental Clinical Oncology, Radiumstationen, Aarhus, Denmark.

## Abstract

Cell survival was investigated in an intraocular retinoblastoma-like tumour 30 min to 48 h after photodynamic therapy. The survival of the cells was assessed by an in vivo to in vitro colony forming assay, estimated by either the plating efficiency of the treated tumour cells compared to non-treated cells or the number of clonogenic cells per mg excised tumour. Curves showing cell survival as a function of the time between light irradiation and excision of the intraocular tumours were biphasic. This suggests more than one PDT tissue destruction mechanism in vivo (i.e. an early direct cell damage plus a subsequent late damage occurring in the tumour tissue left in situ after treatment). The delayed mechanism may be due to changes in the environment of the tumours probably caused by vascular damage. Tumour cells sensitised by Photofrin II in vivo and excised from the eyes were damaged by light when irradiated in vitro and this was dependent on the light energy dose. This showed that cellular Photofrin II uptake in the eye tumours was sufficient for direct cell damage and thus supports the suggestion that direct and indirect tumour destruction occurs in this eye tumour after photodynamic therapy.


*Br. J. Cancer* (1989), **59**, 869–872 © The Macmillan Press Ltd., 1989

### Photodynamic therapy effect in an intraocular retinoblastoma-like tumour assessed by an *in vivo* to *in vitro* colony forming assay

J. Winther

*Danish Cancer Society, Department of Experimental Clinical Oncology, Radiumstationen and Department of Ophthalmology, Aarhus University Hospital, DK-8000 Aarhus C, Denmark.*

**Summary** Cell survival was investigated in an intraocular retinoblastoma-like tumour 30 min to 48 h after photodynamic therapy. The survival of the cells was assessed by an *in vivo* to *in vitro* colony forming assay, estimated by either the plating efficiency of the treated tumour cells compared to non-treated cells or the number of clonogenic cells per mg excised tumour. Curves showing cell survival as a function of the time between light irradiation and excision of the intraocular tumours were biphasic. This suggests more than one PDT tissue destruction mechanism *in vivo* (i.e. an early direct cell damage plus a subsequent late damage occurring in the tumour tissue left *in situ* after treatment). The delayed mechanism may be due to changes in the environment of the tumours probably caused by vascular damage. Tumour cells sensitised by Photofrin II *in vivo* and excised from the eyes were damaged by light when irradiated *in vitro* and this was dependent on the light energy dose. This showed that cellular Photofrin II uptake in the eye tumours was sufficient for direct cell damage and thus supports the suggestion that direct and indirect tumour destruction occurs in this eye tumour after photodynamic therapy.

Numerous experimental and clinical studies have resulted from the interest in photodynamic therapy (PDT) as a selective therapeutic modality in the management of cancer. The principle of the treatment involves the combined actions of three agents, i.e. haematoporphyrin derivatives, visible light and oxygen, each of which are relatively non-toxic, but combined together develop severe oxidative damage to surrounding biological substrates (Moan, 1986; Dougherty, 1987).

A number of PDT studies on experimental eye tumours in animals or retinoblastomas and choroidal melanomas in humans have been reported in order to evaluate the possibility of using PDT for management of intraocular tumours (Bruce, 1984; Franken *et al.*, 1985; Gomer *et al.*, 1985; Murphree *et al.*, 1987; Ohnishy *et al.*, 1986; Sery *et al.*, 1987; Winther *et al.*, 1988). The advantage of PDT in this approach is that it is possible to focus the light directly through the pupils and onto the visible tumour surfaces.

At the present time histopathological intraocular tumour damage, significant growth delay and tumour cure have been demonstrated in retinoblastomas following PDT (Winther & Ehlers, 1988; Horsman & Winther, 1989). However, tumour recurrences also have been reported, indicating that a more basic knowledge of the mechanism of PDT is mandatory in order to improve the anti-tumour effect (Winther & Overgaard, 1989).

The aim of the current study has thus been to describe the PDT anti-tumour effect by characterisation of the kinetics of cell damage following treatment *in vivo*. This paper focuses on the occurrence of very early cell damage as well as effects appearing later during the first two days after treatment.

### Materials and methods

### *Retinoblastoma-like tumour model*

The retinoblastoma-like cell line, EXP-5 (Kobayashi *et al.*, 1982) was used for producing intraocular tumours. Cells were inoculated into the vitreous body of 6–13-day-old inbred F344 rats producing solid tumours, which are supplied with blood vessels from the retina. The *in vivo* and *in vitro* growth characteristics, histopathology, cytogenetics and flow cytometry have been previously described (Winther, 1986; Winther *et al.*, 1987).

Received 15 March 1988, and in revised form, 24 January 1989.

### *Haematoporphyrin derivative*

The purified haematoporphyrin derivative, designated Photofrin II (Photomedica, NJ, USA) was used for all experiments. It was stored in darkness at −20°C until use. After thawing the drug was diluted in saline and injected intraperitoneally into animals, 24 h before light irradiation.

### *Light irradiation and light dosimetry*

*In vivo* The rats were anaesthetised by pentobarbital and the pupil was dilated by one drop of tropicamid 1% before light irradiation.

The tumours were irradiated by a 50 mW helium-neon laser (NEC, Japan) delivering light with a wavelength of 632.8 nm. The laser beam was expanded by a −2 diopter lens 26 cm in front of the eye allowing an area of 0.6 $cm^2$ of the eye to be irradiated, which resulted in an apparently homogeneous light spot. The energy of the laser was measured by a laser powermeter (Phir Laser Power monitor, Ophir Optica Ltd, Jerusalem, Israel). The fluence rate on the cornea was 50 $mW\,cm^{-2}$ giving a fluence of 3 $J\,cm^{-2}\,min^{-1}$.

The tumours were exposed to light either *in vivo* before excision of the tumours or in tissue culture flasks after plating.

*In vitro* Tissue culture flasks containing retinoblastoma-like cells were light irradiated in a waterbath at 26–30°C by three 3,000 W linear xenon flash lamps filtered by acrylic cut-off-filters passing light in the range of 600–700 nm. A dispersing fat emulsion was added to the water to obtain an approximately homogeneous light irradiation (Bjerring *et al.*, 1987). The light energy was measured on the bottom of the flasks by a high precision photometer with a cosine correction (Brüel & Kjær, Copenhagen, Denmark). The fluence rate was 29 $mW\,cm^{-2}$, giving a fluence of 1.74 $J\,cm^{-2}\,min^{-1}$.

In vivo *to* in vitro *colony forming assay* The tumour size was assessed by a stereoscopic microscope (Winther, 1986). Tumours covering more than half of the retinae were used for experiments. The Photofrin II was injected intraperitoneally 24 h before light irradiation. The animals were anaesthetised with pentobarbital before enucleation. The globes were bisected under a microscope and all visible tumour tissue was removed. Excised tumour was weighed and generally found to be between 50–120 mg. A single cell suspension was prepared from all the removed tumour mass without the use of enzymes by propagating the tissue through needles of gradually reduced diameter.

The total number of morphologically intact cells was counted (i.e. cells having an intact and smooth outline with a bright halo) for calculation of the plating efficiencies (PE).

Cell viability was primarily assessed by the exclusion of trypan blue dye so that the concentration of cells in suspension could be adjusted in order to produce 75–200 colonies per flask. Cell damage according to the trypan blue exclusion test increased according to the treatment and typically varied between 5–10% in non-treated tumours and up to 90–95% in tumours treated with 5 or 10 mg Photofrin II and left *in situ* for 48 h.

All tumour cells were diluted in a known amount of RPMI 1640 tissue culture medium supplemented with 15% fetal calf serum and containing $0.1\,mg\,ml^{-1}$ streptomycin, $100{,}000\,IU\,ml^{-1}$ penicillin and $2\times10^5$ feeder cells (heavily irradiated retinoblastoma-like cells) as previously described (Winther, 1989). The cells were incubated in 5% $CO_2$ and 95% air at 37°C for 9–11 days. Colonies were then fixed on the bottom of the flasks with methanol and stained with toluidine blue. Those colonies containing more than approximately 50 cells were counted using a microscope. The PE of non-treated tumour cells (i.e. the percentage of the total number of plated cells forming colonies after explanting into the tissue culture flasks) varied from 10 to 40% in the present experiments.

*Evaluation of results*

*In vivo to in vitro colony forming assay* The therapeutic response was assessed from the *relative* plating efficiency, i.e. $100\times PE_{treated}/PE_{untreated}$ (Figure 1) and from the *relative* number of clonogenic cells per mg tumour tissue (Figure 2). The relative number of clonogenic cells per mg tumour tissue was the number of cells in treated tumours compared to the number in non-treated controls. Clonogenicity in each tumour was calculated from the number of colonies counted in 3–9 flasks. Each point on the curves represents the mean of 8–14 tumours. Logarithms of the observed number of clonogenic cells showed a normal distribution. The logarithmic mean of each group was tested by Student's *t* test. Table I shows the total number of clonogenic cells per mg tumour tissue in the treated eyes.

*In vitro* The PDT effect on tumour cells sensitised by Photofrin II *in vivo* followed by light irradiation *in vitro* in the culture flasks was described in terms of the relative plating efficiency as defined above. The PDT sensitivity was described by $D_0$ from the survival curves (i.e. the light energy dose required for inactivation of $1-1/e$ of the cells). All *in vitro* experiments were performed at least three times on different days. Each data point was calculated from 3–9 flasks in each experiment.

### Results

The cell survival of intraocular tumours assessed by an *in vivo* to *in vitro* assay at variable time after different treatment doses and expressed as relative PE is shown in Figure 1. The survival curves for 5 and $10\,mg\,kg^{-1}$ Photofrin II appeared to have a biphasic pattern as a function of the time the tumours were left in the eyes after treatment, with the slopes being steeper in the first 30 min.

The data of Figure 1 were also expressed as the number of relative clonogenic cells per mg treated tumour in Figure 2. These results also demonstrated a biphasic curve of cell survival similar to the patterns shown in Figure 1. The curves declined rapidly, and a statistically significant decrease was shown 30 min after the light irradiation for the groups treated with either 10 or $5\,mg\,kg^{-1}$ Photofrin II ($P<0.001$). A borderline statistical significance was also demonstrated in animals treated with $2.5\,mg\,kg^{-1}+90\,J\,cm^{-2}$ ($P=0.07$). The mean and the range of the total number of cells per mg tumour tissue are shown in Table I.

**Figure 1** Relative plating efficiency in PDT treated tumours compared to non-treated tumours. Each data point represents the mean of 8–14 tumours. Bars are s.e. and were deleted from some points for clarity.

**Figure 2** Percentage of clonogenic cells per mg tumour tissue in PDT treated tumours compared to non-treated tumours. Each data point represents the means of 8–14 tumours.

In order to investigate whether the retinoblastoma-like cells sensitised with Photofrin II *in vivo* had taken up sufficient drug for cellular damage, the animals were injected with Photofrin II *in vivo*. The tumours were excised and explanted in culture flasks 24 h after drug administration. Single cells were then exposed to light in the flasks. Survival

**Table I** The total number of clonogenic cells per mg tumour tissue in the retinoblastoma-like tumours 30 min to 48 h after PDT

| *Photofrin II* (*mg kg*$^{-1}$) | *Light* (*J cm*$^{-2}$) | *0.5 h* (*cells*) | *4 h* (*cells*) | *24 h* (*cells*) | *48 h* (*cells*) |
|---|---|---|---|---|---|
| 10 | 45 | 1,471[a] (481–5,916) | 838 (226–1,950) | 472 (0–4,898) | 96 (0–1,059) |
| 5 | 90 | 1,823 (360–6,577) | 1,268 (141–3,681) | 422 (16–2,729) | 112 (0–671) |
| 2.5 | 90 | 2,722 (410–7,798) | 1,474 (472–3,724) | 2,350 (395–6,603) | 1,950 (63–6,124) |
| 2.5 | 270 | – | – | 500 (0–2,501) | – |
| 0 | 0 | 4,761 (1,011–18,858) | – | | – |

[a]Mean values with range shown in parentheses.

**Figure 3** Survival of tumour cells sensitised to Photofrin II *in vivo* 24 h before excision and plating as single cells in tissue culture flasks for colony growth. The cells were exposed to light *in vitro*. The curves were fitted by linear regression analysis. Each data point was calculated from 3–9 flasks and all experiments were repeated on at least three different days. Bars are s.e. $D_0$ was calculated to 20 J cm$^{-2}$ for 20 mg kg$^{-1}$ Photofrin II, 30 J cm$^{-2}$ for 10 mg kg$^{-1}$ Photofrin II and 92 J cm$^{-2}$ for 5 mg kg$^{-1}$ Photofrin II.

curves of the tumour cells from animals treated with 5, 10 or 20 mg kg$^{-1}$ Photofrin II are shown in Figure 3. All three curves were statistically different and indicated an increased cellular Photofrin II concentration *in vivo* at large treatment doses in the range of 5–20 mg kg$^{-1}$. The surviving fraction of cells treated with 2.5 mg kg$^{-1}$ Photofrin II followed by a light exposure of 69 J cm$^{-2}$ was 78% (data not shown), suggesting a low cellular drug concentration in the cells 24 h after injection.

The survival curve patterns for the present cells sensitised with Photofrin II *in vivo* were similar to those previously reported following *in vitro* sensitisation with Photofrin II using the same light irradiation equipment and fluence rate (Winther, 1989).

### Discussion

There is controversy as to the importance of direct cell damage in the response of tumours to PDT. Henderson *et al.* (1985) reported that early cell damage did not occur in subcutaneously growing experimental mice tumours (EMT-6 and RIF-1 tumours) assessed by an *in vivo* to *in vitro* clonogenic assay. The results from that study combined with several studies on vascular damage, haemorrhagic necrosis and blood flow changes after PDT (Bugelski *et al.*, 1981; Selman *et al.*, 1984; Berenbaum *et al.*, 1986; Star *et al.*, 1986; Nelson *et al.*, 1987) have consequently been the fundamental basis for regarding vascular damage as the main mechanism for PDT damage *in vivo*.

However, our current investigation, also using the *in vivo* to *in vitro* assay procedure, demonstrated biphasic decline in cell survival in the intraocular tumours as a function of time after the light irradiation. This suggests the involvement of more than one mechanism, one appearing early after treatment and the other later. The early cell damage occurring within 30 min after light irradiation resulted in a survival level of 30–57% of the total numbers of cells per mg tumour tissue while the late effect inactivated the cells down to the 1% survival level (Table I).

In the current intraocular tumour, PDT has previously been shown to damage the vascular system and to decrease blood flow to about 25% of the normal level 24 h after light administration (Winther & Ehlers, 1988; Horsman & Winther, 1989). Tumour destruction by the late effect can thus be explained by tissue anoxia *in vivo*. On the other hand, the early cellular damage demonstrated within 30 min after the light irradiation could not be explained by tissue anoxia because the blood flow in the treated tumours did not start decreasing until at least 4 h after the light irradiation (Horsman & Winther, 1989).

The survival curves of retinoblastoma-like cells sensitised by Photofrin II in the eyes followed by light irradiation *in vitro* after the tumours were excised (Figure 3) confirmed that the uptake of Photofrin II into cells was related to the drug dose administered. Furthermore, the cellular uptake of Photofrin II was sufficient for PDT cell destruction.

The results in the current study are thus indicative of involvement of a direct cell damage mechanism following PDT *in vivo*. The results showed the ratio between cell survival assessed by the relative plating efficiencies and the relative number of clonogenic cells/mg tumour was in the range of 1.1–1.5 after 30 min, increasing to 1.6–6.0 48 h after the light irradiation (Figures 1 and 2). This discrepancy can be explained by a treatment-induced early cell loss resulting in a lower cell yield and a consequently artificially increased PE in the treated tumours.

Such differences between surviving fractions and number of clonogenic cells have also been previously reported following hyperthermia treatment of experimental tumours (Marmor *et al.*, 1977). This supports the hypothesis that

PDT and local hyperthermia treatment have some similar tissue destruction mechanisms (Waldow & Dougherty, 1984).

The difference in number of surviving cells between non-treated tumours and tumours 30 min after treatment was more than 50% when 5 or $10 \text{mg kg}^{-1}$ Photofrin II was administered.

This was conflicting data compared to the lack of early cell damage in the experimental mice tumours reported by Henderson *et al.* (1985). Such differences are difficult to explain because in both studies the tumours were treated with similar external light irradiation and Photofrin II doses. However, the discrepancies may be due to light attenuation passing through the skin, which did not occur passing through the transparent cornea and lens. This suggests that the retinoblastoma-like cells received a relatively higher energy dose than the subcutaneous tumours. The diverging results may also be explained by differences in cellular Photofrin II uptake due to varying degrees of tumour vascularity. This is favoured by previously reported observations of higher concentration of tritium-labelled haematoporphyrin derivatives close to tumour blood vessels and a decline in the concentration at large distances from the vessels (Bugelski *et al.*, 1981). A more efficient PDT tumour response may then hypothetically be obtained by modified photosensitisers which allow a more homogeneous tissue distribution and/or a higher degree of cellular uptake.

In conclusion, the anti-tumour effect of PDT in the present intraocular tumour consists of an early direct cell inactivation plus a secondary tissue damage due to changes in the tumour environment which probably is due to vascular damage. Nevertheless, it is important to emphasise that conclusive evidence of anoxic tissue damage following PDT has not been presented so far.

The retinoblastoma-like cell line (EXP-5 cells) was kindly supplied by Dr N. Mukai, Eye Research Institute of Retina Foundation, Boston, MA, USA. The study was supported by The Danish Cancer Society, Landsforeningen til Bekæmpelse af Øjensygdomme og Blindhed, Astrid Thaysen Foundation, and Direktør Jacob Madsen and Olga Madsens Foundation. The author wishes to thank Ms L. Christensen and Ms M. Thomsen for expert technical assistance and Photomedica for supply of Photofrin II.

### References

BERENBAUM, M.C., HALL, G.W. & HAYES, A.D. (1986). Cerebral photosensitization by hematoporphyrin derivative. Evidence for an endothelial site of action. *Br. J. Cancer,* **53,** 81.

BJERRING, P. (1987). Intratumoural light distribution in an experimental mouse tumour by a diffuse-light irradiator compared with unilateral helium-neon light for photodynamic therapy. *Int. J. Radiat. Biol.,* **52,** 191.

BRUCE, R.A. (1984). Evaluation of hematoporphyrin photoradiation therapy to treat choroidal melanomas. *Laser Surg. Med.* **4,** 59.

BUGELSKI, P.J., PORTER, C.W. & DOUGHERTY, T.J. (1981). Autoradiographic distribution of hematoporphyrin derivative in normal and tumour tissue of the mouse. *Cancer Res.,* **41,** 4606.

DOUGHERTY, T.J. (1987). Photosensitizers: therapy and detection of malignant tumours. *Photochem. Photobiol.,* **45,** 679.

FRANKEN, K.A.P., DELFT, J.L., DUBBELMANN, T.M. and 4 others (1985). Hematoporphyrin derivative photoradiation treatment of experimental malignant melanoma in the anterior chamber of the rabbit. *Curr. Eye Res.,* **4,** 641.

GOMER, C.J., JESTER, J.V., RAZUM, N.R., SZIRTH, B.S. & MURPHREE, A.L. (1985). Photodynamic therapy on intraocular tumours: examination on hematoporphyrin derivative distribution and long-term damage in rabbit occular tissue. *Cancer Res.,* **45,** 3718.

HENDERSSON, B.W., WALDOW, S.M., MANG, T.S., POTTER, W.R., MALONE, P.B. & DOUGHERTY, J. (1985). Tumor destruction and kinetics of tumor cell death in two experimental mouse tumours following photodynamic therapy. *Cancer Res.,* **45,** 572.

HORSMAN, M.R & WINTHER, J. (1989). Vascular effects of photodynamic therapy in an intraocular retinoblastoma-like tumour. *Acta Oncol.* (in the press).

KOBAYASHI, M., MUKAI, N., SOLISH, S.P. & POMEROY, M.E. (1982). A highly predictable animal model of retinoblastoma. *Acta Neuropathol.,* **57,** 203.

MARMOR, J.B., HAHN, N. & HAHN, G.M. (1977). Tumour cure and cell survival after localized radiofrequency heating. *Cancer Res.,* **37,** 879.

MOAN, J. (1986). Porphyrin photosensitization and phototherapy. *Photochem. Photobiol.,* **43,** 681.

MURPHREE, A.L., COTE, M. & GOMER, C.J. (1987). The evolution of photodynamic therapy techniques in the treatment of intraocular tumours. *Photochem. Photobiol.,* **46,** 919.

NELSON, J.S., LIAW, L.H. & BERNS, M.W. (1987). Tumor destruction in photodynamic therapy. *Photochem. Photobiol.,* **46,** 829.

OHNISHY, Y., YAMANA, Y. & MINEI, M. (1986). Photoradiation therapy using argon laser and a hematoporphyrin derivative for retinoblastoma – a preliminary report. *Jpn. J. Ophthalmol.,* **30,** 409.

SERY, T.W., SHIELDS, J.A., AUGSBURGER, J.J. & SHAH, H.G. (1987). Photodynamic therapy on human ocular cancer. *Ophthalmic Surg.,* **18,** 413.

SELMAN, S.H., KECK, R., KLAUNIG, J.E., KREIMER-BIRNBAUM, M., GOLDBLAT, P.J. & BRITTON, S.L. (1984). Bloodflow in transplantable bladder tumors treated with hematoporphyrin derivative. *Cancer Res.,* **46,** 2532.

STAR, W.M., MARIJNISSEN, H.P., VAN DEN BERG BLOK, A.E., VERSTEEG, J.A., FRANKEN, K.A. & REINHOLD, H.S. (1986). Destruction of rat mammary tumor and normal tissue microcirculation by hematoporphyrin derivative photoradiation *in vivo* in sandwich observation chambers. *Cancer Res.,* **46,** 2532.

WALDOW, S.M. & DOUGHERTY, T.J. (1984). Interaction of hyperthermia and photoradiation therapy. *Radiat. Res.,* **97,** 380.

WINTHER, J. (1986). *In vitro* and *in vivo* growth of an intraocular retinoblastoma-like tumour in F344 rats. *Acta Ophthalmol.,* **64,** 657.

WINTHER, J., JENSEN, O.A., PRAUSE, J.U. & TOMMERUP, N. (1987). Characterization of an intraocular retinoblastoma-like tumour. *Acta Ophthalmol.,* **65,** 491.

WINTHER, J. & EHLERS, N. (1988). Histopathological changes in an intraocular retinoblastoma-like tumour following photodynamic therapy. *Acta Ophthalmol.,* **66,** 69.

WINTHER, J. EHLERS, N. & OVERGAARD, J. (1988). The effect of photodynamic therapy alone and combined with misonidazole of X-rays for management of an intraocular retinoblastoma-like tumor. *Photochem. Photobiol.,* **47,** 419.

WINTHER, J. (1989). Effect of Photofrin II and light energy on retinoblastoma-like cells *in vitro* – dose–response relationships and recovery ratios. *J. Can. Res. Clin. Oncol.,* **115,** 73.

WINTHER, J. & OVERGAARD, J. (1989). Dose–response relationships in photodynamic therapy on intraocular retinoblastoma-like tumours and normal eye tissues. *Acta Ophthalmol.* (in the press).